# A phase 1 study of the safety, tolerability, pharmacodynamics, and pharmacokinetics of tenapanor in healthy Japanese volunteers

**DOI:** 10.1007/s10157-016-1302-8

**Published:** 2016-07-01

**Authors:** Susanne Johansson, David P. Rosenbaum, Mikael Knutsson, Maria Leonsson-Zachrisson

**Affiliations:** 1 AstraZeneca Gothenburg, Mölndal, Sweden; 2 Ardelyx, Inc., 34175 Ardenwood Blvd, Fremont, CA 94555 USA

**Keywords:** Tenapanor, Sodium absorption, Phosphate absorption, Sodium, dietary, NHE3 protein, Chronic kidney disease

## Abstract

**Background:**

Tenapanor (RDX5791, AZD1722), a small molecule with minimal systemic availability, is an inhibitor of the sodium/hydrogen exchanger isoform 3 (NHE3). Tenapanor acts locally in the gut to reduce absorption of sodium and phosphate. It is being developed for the treatment of patients with hyperphosphatemia in CKD requiring dialysis and patients with constipation-predominant irritable bowel syndrome. We report the safety, pharmacodynamics, and pharmacokinetics of tenapanor in Japanese volunteers.

**Methods:**

In this phase 1, double-blind study (NCT02176252), healthy Japanese adults (aged 20–45 years) received single-dose tenapanor 180 mg (*n* = 6), repeated-dose tenapanor 15, 30, 60, or 90 mg twice daily (*n* = 12 each) for 7 days, or placebo (*n* = 14). All participants received a standardized diet.

**Results:**

Single and repeated doses of tenapanor resulted in higher mean stool sodium content vs. placebo (single dose, 41.9 mmol/day; repeated dose, range of means 21.3–32.2 mmol/day; placebo, 4.1 mmol/day) accompanied by lower urinary sodium content (single dose, 110 mmol/day; repeated dose, 101–112 mmol/day; placebo, 143 mmol/day). Additionally, stool phosphorus content was increased (single dose, 31.0 mmol/day; repeated dose, 17.6–24.8 mmol/day; placebo, 16.8 mmol/day) and urinary phosphorus content decreased (single dose, 18.7 mmol/day; repeated dose, 15.3–19.4 mmol/day; placebo, 25.5 mmol/day). Tenapanor had minimal systemic exposure, provided a softer stool consistency, and was well tolerated.

**Conclusions:**

Tenapanor treatment reduced absorption of intestinal sodium and phosphate from the gut in Japanese adults. Tenapanor had minimal systemic exposure and was well tolerated. Further research into the clinical benefits of tenapanor is warranted.

**Electronic supplementary material:**

The online version of this article (doi:10.1007/s10157-016-1302-8) contains supplementary material, which is available to authorized users.

## Introduction

Members of the sodium/hydrogen exchanger (NHE) family of proteins, which are expressed throughout the gastrointestinal tract, facilitate the electro-neutral exchange of sodium ions for intracellular hydrogen ions across membranes [1]. NHE isoform 3 (NHE3) is particularly important for intestinal sodium transport and subsequent fluid homeostasis [2–4].

Tenapanor (RDX5791, AZD1722), a small molecule with minimal systemic availability, is an inhibitor of NHE3 that acts locally in the gut [5]. Preclinical and early clinical studies show that tenapanor reduces absorption of sodium [5] and phosphate [6, 7] from the gut, with a concomitant increase in stool fluid content. Sodium/fluid and phosphate imbalances play a role in many conditions, such as hypertension, heart failure, and chronic kidney disease (CKD) [8–14], and stool consistency is of relevance in constipation-related disorders.

In an animal model of CKD (5/6 nephrectomized rats fed a high-salt diet), tenapanor treatment provided protective effects on renal function and reduced extracellular fluid volume and blood pressure [5]. In addition, an animal model of uremic calcification demonstrated that tenapanor treatment reduced vascular calcification [6]. In healthy volunteer studies, tenapanor treatment over 7 days reduced sodium absorption, resulting in increases in stool sodium content of up to 50 mmol (2.9 g table salt) per day [5].

The most recent guidelines on the treatment of patients with CKD from the Japanese Society of Nephrology (published in 2013) recommend restriction of dietary sodium intake to 2.4 g (approximately 6 g table salt) per day [15], and the American Heart Association recommends restriction of sodium intake to 1.5 g (3.8 g table salt) per day in the general population [11]. There are currently no approved drugs that reduce sodium absorption. Therefore, the only means by which sodium uptake can be controlled is by diet, which is often ineffective.

Tenapanor is being evaluated for the treatment of patients with hyperphosphatemia in CKD requiring dialysis and patients with constipation-predominant irritable bowel syndrome (IBS). As a component of its clinical development program, it is important to evaluate the clinical pharmacology of tenapanor in Japanese individuals, so that the agent may be made available for the treatment of patients with these conditions in Japan in the future. Here, we report data from the first study of tenapanor in a Japanese population. This study evaluated the safety, tolerability, pharmacodynamics, and pharmacokinetics of tenapanor in healthy volunteers.

## Materials and methods

### Study design

This was a phase 1, double-blind, randomized, placebo-controlled study in healthy Japanese adult volunteers (ClinicalTrials.gov identifier: NCT02176252). A group of healthy Caucasian individuals was included, primarily to collect further safety data on tenapanor at the highest dose in Caucasian people. The study was conducted at a clinical pharmacology unit (CPU) at WCCT Global, Cypress, CA, USA. The study protocol and consent forms were reviewed and approved by the Institutional Review Board Aspire IRB, Santee, CA, USA (approval number D5611C00005) before commencement of the study.

After screening, eligible participants were admitted to the CPU 2 days before assignment to treatment. Japanese participants were allocated sequentially in the order of completion of their screening assessments to groups evaluating tenapanor hydrochloride (hereafter referred to as tenapanor) given as a single 180 mg dose or one of four repeated doses (tenapanor 15, 30, 60, or 90 mg twice daily over 7 days). Within the single-dose group, participants were randomly assigned to tenapanor (180 mg) or placebo in a 3:1 ratio. Within the repeated-dose groups, participants were randomly assigned to tenapanor or placebo, all twice daily for 7 days, in a 4:1 ratio. Caucasian participants were randomly assigned to either tenapanor 90 mg or placebo, both twice daily for 7 days, in a 4:1 ratio. Data from the single-dose group was obtained before commencing repeated dosing. The repeated-dose 15–60 mg twice-daily groups were run in parallel to each other. All safety data were evaluated before dose escalation to the highest repeated dose of 90 mg twice daily.

A follow-up visit was scheduled for each participant 7–10 days after discharge from the CPU (on day 2 for the single-dose group and day 8 for the repeated-dose groups).

### Participants

This study was conducted in accordance with the Declaration of Helsinki. All participants provided written informed consent. Men and women could be included if they were aged 20–45 years with a body mass index of 19–27 kg/m^2^ and a weight of 45–100 kg. All participants had to be healthy, as judged by their medical history, laboratory screening, physical examination, and 12-lead paper electrocardiography (ECG). To be considered Japanese, both of an individual’s parents and both pairs of grandparents had to be Japanese, and the individual must have been born in Japan and must not have lived outside Japan for more than 5 years.

Individuals were excluded if they: had a history or presence of gastrointestinal, hepatic, or renal disease, including gastrointestinal surgery (other than appendectomy) or any other condition known to interfere with absorption, distribution, metabolism, or excretion of drugs; had loose stools (Bristol Stool Form Scale [BSFS] [16] score of 6 or 7) for 2 or more days in the 7 days before randomization; had used medications known to affect stool consistency and/or gastrointestinal motility during the 7 days before assignment to treatment; or had used electrolyte supplements containing sodium, potassium, chloride, or bicarbonate during the 7 days before assignment to treatment. All participants were expected to abstain from taking any medication (prescribed or over-the-counter products), other than paracetamol/acetaminophen, hormone-replacement therapy and oral contraceptives, from 2 weeks before the first administration of investigational product until after the follow-up visit.

### Study drug administration

Tenapanor was available in 15, 30, or 60 mg non-formulated capsules. In the single-dose (180 mg) group, tenapanor or corresponding placebo capsules were administered orally 5–10 min before breakfast. In the repeated-dose groups, tenapanor or corresponding placebo capsules were administered orally 5–10 min before breakfast and dinner.

While in the CPU, including during the 2-day run-in period, participants received a standardized diet with an approximate sodium content of 4.5 g (195 mmol) per day [1.5 g (65 mmol) in each of three daily meals], equivalent to 11.4 g of table salt per day. Phosphate content of meals was not standardized; however, all individuals received the same meals on the same study days, thereby facilitating consistent phosphate intake. Participants did not have access to table salt or sodium-containing sauces and spices during meals. Fluid intake was ad libitum.

### Study assessments

Adverse events were recorded throughout the study. Vital signs were recorded before the first dose of study drug, on study days 1 and 2 (all treatment groups), and on day 8 (repeated-dose groups). Physical examinations were conducted at screening, on study day 1 (all groups), on day 8 (repeated-dose groups), and at the follow-up visit (all groups). Digital ECG measurements were recorded on study days 1 and 2 (all groups) and days 7 and 8 (repeated-dose groups). Safety laboratory evaluations (fasting state) were made on study day 1 (all treatment groups), day 2 (single-dose group), and day 8 (repeated-dose groups).

Pharmacodynamic measures included daily stool and urinary sodium and phosphorus content and daily stool frequency, weight, and consistency (as measured by BSFS score on the 7-point scale from type 1 [hard lumps] to type 7 [watery] [16]). Bowel movement assessments were performed and daily urine samples collected from the day before assignment to treatment (day −1) to the day after receiving the last dose. The daily collection intervals began after the morning dose on one day and ended just before the morning dose on the next day; for days when no dose was administered, the time that the morning dose would have been administered was used.

Plasma concentrations of tenapanor were determined from blood samples collected pre-dose and 1, 2, 4, 8, and 24 h post-dose on day 1 for the single-dose group, and pre-dose and 1, 2, 4, and 8 h after the morning dose on days 1 and 7 for the repeated-dose groups.

### Analytical methods

Plasma concentrations of tenapanor were measured by MicroConstants, Inc. (San Diego, CA, USA). Human plasma samples containing tenapanor, its deuterated analog as the internal standard, and dipotassium ethylenediaminetetraacetic acid as the anticoagulant were processed first by protein precipitation followed by back extraction into an acidic aqueous solution. The extracts were injected and analyzed by reversed-phase high-performance liquid chromatography using a Synergi Hydro-RP column (Phenomenex, Torrance, CA, USA) maintained at 35 °C. The mobile phase was nebulized using heated nitrogen in a Z-spray source/interface set to electrospray positive-ionization mode. The ionized compounds were quantified using mass spectrometry (MS/MS). The lower and upper limits of quantification were 0.5 and 500 ng/mL, respectively. The accuracy (variation in measured concentration compared with theoretical concentration) and precision (coefficient of variation [SD/mean] within replicates) of quality-control standards of tenapanor were determined at concentrations of 0.5, 1.5, 20, and 400 ng/mL. Accuracy and precision were in the ranges of −1.5 to 8.0 and 2.8–9.1 %, respectively. Precision and accuracy no greater than 20 % were required for quality-control standards at the lower limit of quantification.

Sodium and phosphorus content of stool samples was determined by RTI International (Research Triangle Park, NC, USA). Stool samples were transferred to the laboratory in a frozen state and stored at −20 °C. Samples were partially digested in approximately twice their weight of concentrated nitric acid and then heated at 60 °C for 3 h; aliquots of the resulting partially digested samples (5 mL) were further digested with nitric and hydrochloric acids and diluted to 50 mL with deionized water, before centrifugation to precipitate insoluble matter. The sodium and phosphorus content of the supernatant liquid was measured using inductively coupled plasma-optical emission spectrometry (iCAP 6500 ICP-OES analyzer, Thermo, USA). The validated lower limit of quantification was defined by the lowest replicate electrolyte concentration that was measured with acceptable accuracy (±20 % of the nominal back-calculated concentration from the calibration curve). At least four of six quality-control standards had to be quantified as within 20 % of their theoretical value for an analysis run to be acceptable. The overall precision and overall bias (a measure of accuracy) of the assay for sodium, as determined by analysis of quality-control standards were in the ranges of 3.2–5.8 % and 2.9–14.5 %, respectively. The overall precision and overall bias of the assay for phosphorus were in the ranges of 1.9–3.3 and 0.4–3.8 %, respectively.

Sodium and phosphorus content of urine samples were determined using ion selective electrode measurements by standard clinical laboratory techniques (Consolidated Medical Bioanalysis, Cypress, CA, USA).

### Statistical analyses

Pharmacodynamic measurements were summarized according to dose group using descriptive statistics, with calculations of daily means and standard deviations across the treatment period. For each participant, mean on-treatment daily stool and urinary sodium and phosphorus content and daily stool frequencies were calculated as the mean of all available measurements following assignment to treatment divided by the number of days of treatment for which measurements were available. For each individual, mean BSFS score and mean stool weight were calculated as the mean for each 24-h period, and the 24-h means over the full treatment period were used to provide the mean daily BSFS score and mean daily stool weight. Statistical analyses were conducted using SAS version 9.2 (SAS Institute, Carey, NC, USA).

## Results

### Study participants

Of the 144 people screened, 83 healthy Japanese and Caucasian individuals were assigned to treatment. The demographic and baseline characteristics of the participants were generally well balanced across the treatment groups (Table 1). The study population included 68 Japanese individuals. Of these, 65 completed the study: two individuals (one each from the single-dose tenapanor 180 mg group and the repeated-dose tenapanor 15 mg twice-daily group) withdrew from the study during the follow-up period, both due to relocation, after having received all specified doses of tenapanor according to protocol. A third Japanese individual in the placebo group withdrew 3 days after randomization (participant decision). There were 15 Caucasian individuals who were assigned to treatment (12 to tenapanor and three to placebo), all of whom completed the study.

**Table 1:** Demographic and baseline characteristics of the Japanese participants

Characteristic	Single-dose group	Repeated-dose groups^a^				Placebo (*n* = 14)^b^
	Tenapanor 180 mg (*n* = 6)	Tenapanor 15 mg b.i.d. (*n* = 12)	Tenapanor 30 mg b.i.d. (*n* = 12)	Tenapanor 60 mg b.i.d. (*n* = 12)	Tenapanor 90 mg b.i.d. (*n* = 12)	
Age (years)	37.5 ± 8.7	35.4 ± 5.8	33.2 ± 6.7	30.3 ± 5.5	29.3 ± 8.1	30.9 ± 8.0
Men (*n*)	1	7	11	7	9	10
Height (cm)	161 ± 5	167 ± 8	172 ± 7	167 ± 7	171 ± 9	169 ± 8
Weight (kg)	54.8 ± 7.4	62.0 ± 9.2	69.9 ± 9.0	61.8 ± 9.0	66.1 ± 11.7	64.3 ± 8.4
Body mass index (kg/m^2^)	21.2 ± 1.8	22.2 ± 2.1	23.4 ± 2.0	22.1 ± 2.4	22.4 ± 2.6	22.5 ± 2.1
Serum creatinine (mg/dL)	0.77 ± 0.16	0.84 ± 0.14	0.93 ± 0.14	0.73 ± 0.17	0.86 ± 0.17	0.82 ± 0.11

Caucasian individuals receiving tenapanor 90 mg b.i.d (*n* = 12):^a^ age, 33.3 ± 7.4 years; men, *n* = 8; height, 171 ± 9 cm; weight, 70.8 ± 11.0 kg; body mass index, 24.1 ± 2.3 kg/m^2^; serum creatinine, 0.93 ± 0.19 mg/dL

Caucasian individuals receiving placebo (*n* = 3):^a^ age, 27.7 ± 2.1; men, *n* = 2; height, 167 ± 10 cm; weight, 65.3 ± 8.9 kg; body mass index, 23.5 + 3.1 kg/m^2^; serum creatinine, 0.90 ± 0.20 mg/dL

Data are shown as mean ± standard deviation, unless otherwise stated

*b.i.d.* twice daily

^a^Dosing over 7 days

^b^Combined data from Japanese participants receiving single (*n* = 2) and repeated (*n* = 12) doses

### Safety and tolerability

Tenapanor was generally well tolerated. Overall, a total of 16 individuals (11 Japanese and five Caucasian) reported at least one adverse event during the study, irrespective of the relationship to treatment. This included four individuals receiving placebo (three Japanese and one Caucasian). All reported adverse events were mild, with no serious adverse events or discontinuations due to adverse events.

In Japanese individuals, three adverse events in participants receiving tenapanor were judged to be related to treatment by the study investigator. These consisted of two events of diarrhea (60 and 90 mg twice daily, one per group) and one event of nausea (90 mg twice daily). In Caucasian individuals treated with tenapanor 90 mg twice daily, four adverse events were also judged as being related to treatment by the study investigator, consisting of one event of abdominal pain, two events of diarrhea, and one event of headache.

Across all treatment groups, there were no clinically relevant changes noted amongst individuals receiving single- or repeated-dose tenapanor or placebo in laboratory assessments, vital signs, ECG parameters, or physical examinations (see Supplementary Table S1 for data on Japanese individuals from the repeated-dose groups).

### Pharmacodynamics

#### Sodium and phosphorus excretion

**Fig. 1:**
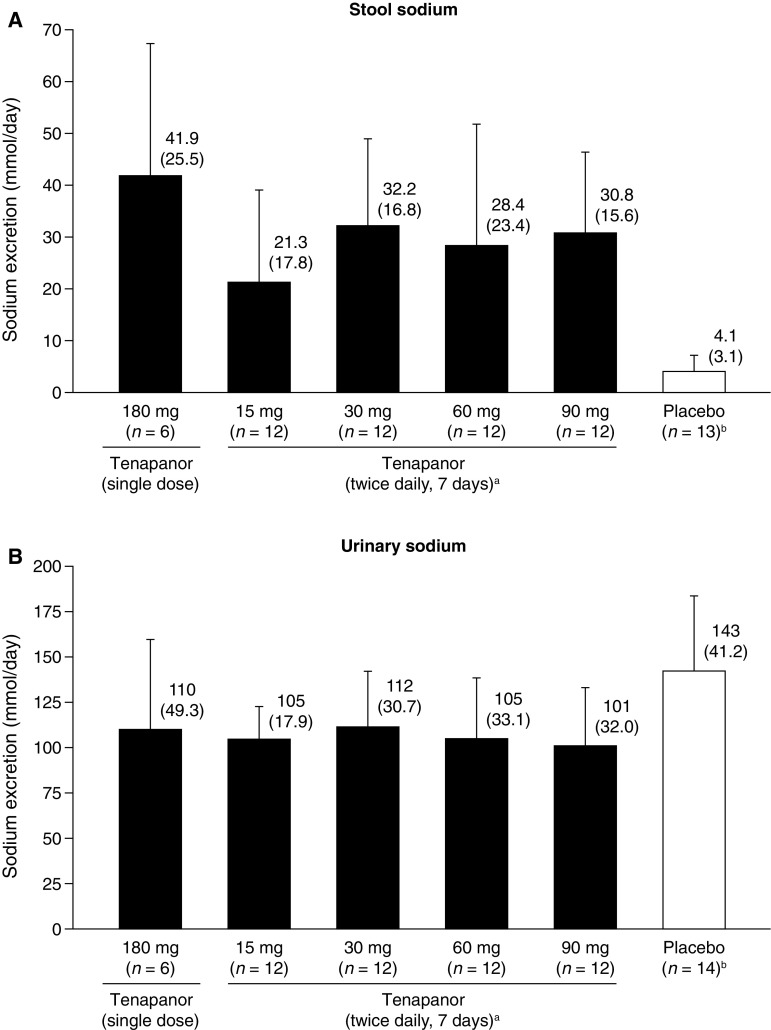
Excretion of sodium via **a** stool and **b** urine in healthy Japanese volunteers treated with tenapanor or placebo. Data are shown as mean + SD; corresponding values given above the bars show mean (SD).  ^*a*^ daily mean over 7 days.  ^*b*^ combined data from participants receiving single (*n* = 2) and repeated (*n* = 12) doses (one individual in the placebo group was excluded from stool analyses owing to a lack of any post-baseline samples). *SD* standard deviation

Stool and urinary sodium content in Japanese individuals following single or repeated doses of tenapanor or placebo are shown in Fig. 1. Twice-daily treatment with tenapanor 15–90 mg for 7 days resulted in mean stool sodium content in the range of 21.3–32.2 mmol/day, with corresponding mean urinary sodium content in the range of 101–112 mmol/day. Variability across the cohorts was high with regard to stool sodium content, with coefficients of variation in the range of 51–84 %; the coefficients of variation were lower for urinary sodium content (17–32 %). Repeated-dose tenapanor resulted in increases in stool sodium content of 17.2–28.1 mmol/day relative to placebo (Fig. 1a) and decreases in urinary sodium content of 31.1–41.4 mmol/day relative to placebo (Fig. 1b). Data from the repeated-dose groups did not support a dose–response relationship with regard to stool and urinary sodium content when data were viewed as the mean across 7 days (as in Fig. 1), or when the data were viewed day-by-day, where the effects of variation are greater (data not shown).

**Fig. 2:**
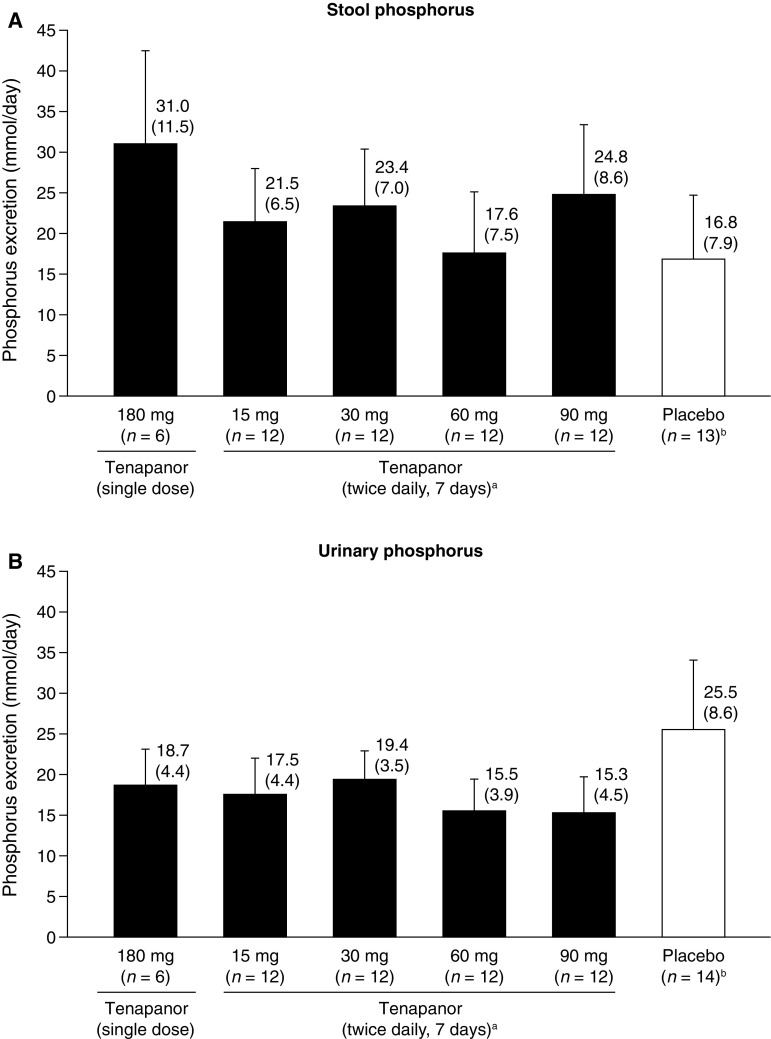
Excretion of phosphorus via **a** stool and **b** urine in Japanese healthy volunteers treated with tenapanor or placebo. Data are shown as mean + SD; corresponding values given above the bars show mean (SD).  ^*a*^ daily mean over 7 days.  ^*b*^ combined data from participants receiving single (*n* = 2) and repeated (*n* = 12) doses (one individual in the placebo group was excluded from stool analyses owing to a lack of any post-baseline samples). *SD* standard deviation

Stool and urinary phosphorus content in Japanese individuals following single- or repeated-dose tenapanor or placebo are shown in Fig. 2. Relative to placebo, repeated-dose tenapanor resulted in increases in stool phosphorus content of 0.8–8.0 mmol/day (Fig. 2a) and decreases in urinary phosphorus content of 6.1–10.2 mmol/day (Fig. 2b). Data from the repeated-dose groups did not support a dose–response relationship with regard to stool and urinary phosphorus content.

In the Caucasian cohort, similar trends of increases in stool sodium and phosphorus content and decreases in urinary sodium and phosphorus content relative to placebo were also observed with repeated-dose tenapanor 90 mg twice daily over 7 days. In individuals receiving tenapanor or placebo, stool sodium content was (mean ± SD) 17.2 ± 10.8 and 3.1 ± 3.7 mmol/day, respectively, and urinary sodium content was 76.6 ± 35.6 and 80.0 ± 22.3 mmol/day, respectively. Corresponding stool phosphorus content was (mean ± SD) 18.8 ± 9.5 and 14.8 ± 5.3 mmol/day, respectively, and urinary phosphorus content was 16.1 ± 3.5 and 21.9 ± 1.6 mmol/day, respectively.

#### Stool frequency, weight, and consistency

The effects of tenapanor on stool frequency, weight, and consistency are shown in Table 2. Twice-daily dosing with tenapanor 15–90 mg resulted in stool frequencies in the range of 1.8–2.1 bowel movements/day, compared with 1.4 bowel movements/day for placebo. An increase in stool weight was also observed, with mean values in the range of 118–134 g/day with tenapanor 15–90 mg, compared with 108 g/day for placebo. Softening of the stool was observed when the participants were treated with tenapanor compared with placebo: mean daily BSFS scores were in the range of 4.4–5.4 in individuals treated with twice-daily tenapanor 15–90 mg, compared with 3.4 for those treated with placebo.

**Table 2:** Summary of stool frequency, weight, and consistency in healthy Japanese individuals treated with tenapanor or placebo

Variable	Single-dose group	Repeated-dose groups^a^				Placebo (*n* = 14)^b^
	Tenapanor 180 mg (*n* = 6)	Tenapanor 15 mg b.i.d. (*n* = 12)	Tenapanor 30 mg b.i.d. (*n* = 12)	Tenapanor 60 mg b.i.d. (*n* = 12)	Tenapanor 90 mg b.i.d. (*n* = 12)	
Stool frequency (bowel movements/day)	3.8 ± 2.3	1.8 ± 1.1	2.1 ± 0.7	1.8 ± 0.7	1.9 ± 0.7	1.4 ± 0.5
Stool weight (g/day)	127 ± 60	118 ± 31	134 ± 42	134 ± 79	132 ± 67	108 ± 47
Stool consistency (mean daily BSFS score)	5.2 ± 2.0	4.4 ± 1.4	5.1 ± 1.3	5.4 ± 0.9	5.4 ± 0.8	3.4 ± 0.5

Data shown are mean ± standard deviation over the treatment period (1 or 7 days for single- or repeated-dose groups, respectively)

*b.i.d.* twice daily, *BSFS* Bristol Stool Form Scale

^a^Dosing over 7 days

^b^Combined data from Japanese participants receiving single (*n* = 2) and repeated (*n* = 12) doses

A similar trend of increased stool frequency and stool weight, accompanied by a softening of stool was also observed in Caucasian individuals, who received tenapanor 90 mg twice daily over 7 days.

### Pharmacokinetics

After treatment with single-dose tenapanor 180 mg, the plasma concentration of tenapanor was below the lower limit of quantification (0.5 ng/mL) in 28 of 30 post-dose plasma samples. Two samples had plasma tenapanor concentrations of 0.58 and 0.70 ng/mL, both of which were taken (from separate individuals) at 4 h post-dose. After repeated twice-daily dosing with tenapanor (15–90 mg) for 7 days, the plasma concentration of tenapanor was below the lower limit of quantification in 539 of 540 post-dose samples. One sample from a Japanese individual receiving tenapanor 60 mg twice daily had a plasma tenapanor concentration of 0.51 ng/mL, which was taken 4 h post-dose on the first day of treatment.

## Discussion

This article reports the first study of Japanese individuals who received tenapanor, a small-molecule inhibitor of NHE3 that acts locally in the gut with minimal systemic drug exposure. Our study, in which participants received a standardized diet, showed that administration of tenapanor over a wide dose range reduced the absorption of dietary sodium and phosphate in Japanese individuals, as demonstrated by increases relative to placebo in stool sodium and phosphorus content and concomitant decreases in urinary sodium and phosphorus content.

With the exception of the lowest dose (15 mg twice daily over 7 days), all other repeated doses of tenapanor (up to 90 mg twice daily over 7 days) resulted in mean daily stool sodium excretion of approximately 30 mmol/day, compared with 4 mmol/day with placebo treatment. Stool sodium excretion of 30 mmol is equivalent to approximately 1.8 g of table salt, or 16 % of the 11.4 g of table salt consumed daily by the study participants. It is important to note, however, that although the mean of stool and urinary electrolyte content was calculated over the whole treatment period, there was large variability in stool sodium content across the cohorts. Less variability across the cohorts was evident with urinary sodium content. Repeated-dose tenapanor resulted in similar stool phosphorus (approximately 18–25 mmol/day) and urinary phosphorus (15–19 mmol/day) content, irrespective of dose. Tenapanor treatment also resulted in increases in stool frequency and weight and a softer stool consistency. The pharmacodynamic data for tenapanor in Japanese individuals are in line with previously reported data in a population of mainly Caucasian and African-American healthy volunteers who received repeated-dose tenapanor: in these individuals, increases in stool sodium [5] and phosphorus [7] content, and decreases in urinary sodium and phosphorus content of broadly similar respective magnitudes, were observed. Consistent with data from the same studies [5], tenapanor was quantifiable in fewer than 1 % of measured serum samples collected from Japanese individuals.

Interestingly, it was noted that in some treatment groups in our study, levels of sodium excreted in stool and urine did not add up numerically to the total sodium intake. While some variability in the collection of urine and stool samples and their analyses could have contributed to this observation, the finding is in line with that of another study conducted in a highly controlled environment, in which sodium excretion at constant sodium intake showed weekly rhythms, resulting in periodic storage of sodium in the body [17].

Excessive sodium intake [8, 11] and fluid overload [10, 18] are well-recognized issues in CKD, with studies in patients on hemodialysis suggesting that reducing sodium intake may lead to a decrease in fluid retention between dialysis sessions [19, 20]. Phosphate retention is part of the complex mineral and bone disorder of CKD and is associated with abnormalities in bone turnover and mineralization and in vascular or other soft-tissue calcification [21]. Hyperphosphatemia is associated with increased mortality in patients with CKD [22–24]. Therefore, reducing sodium and phosphate intake are important components of guidelines for the management of patients with CKD [15, 21, 25].

Our study also showed that repeated-dose tenapanor treatment (up to 90 mg twice daily for 7 days) in Japanese individuals resulted in increases relative to placebo in mean stool phosphorus content that were of a similar magnitude to those observed in a healthy volunteer study of the phosphate binder sevelamer at a dose of 5 g three times daily for 4 days [26]. Tenapanor may, therefore, have potential as a future treatment modality for hyperphosphatemia by improving phosphate control and reducing tablet burden and sodium uptake. The precise mechanism of action of tenapanor with regard to reducing phosphate absorption is currently being evaluated.

Phase 2 trials evaluated the potential clinical benefits of reducing sodium and phosphate absorption by tenapanor treatment in patients with CKD [27–29]. A study in patients with CKD stage 5D (hemodialysis) on the effects of tenapanor on fluid overload [27], and another in patients with type 2 diabetes mellitus and CKD stage 3 on the effect of tenapanor on albuminuria (a marker of renal function decline) [29], did not meet their primary objectives. However, results from a 4-week study in patients with hyperphosphatemia in CKD stage 5D (hemodialysis), show the effects of tenapanor on phosphate absorption translate into clinically meaningful reductions in serum phosphate levels [28].

Tenapanor reduces intestinal sodium absorption, leading to enhanced intestinal fluid volume and transit, as demonstrated by the softer stool consistency and increase in frequency of bowel movements observed in the present study. Tenapanor has also been shown to have an anti-nociceptive effect in an IBS animal model of visceral pain [30], and it is also being evaluated in constipation-predominant IBS [31, 32]. Results from a phase 2b study of tenapanor indicate that these effects translate into clinically meaningful improvements in constipation and abdominal pain in patients with constipation-predominant IBS [33].

The safety and tolerability profiles and pharmacodynamic effects of tenapanor seen in this study in Japanese individuals are consistent with those observed in the Caucasian individuals in this study, and also in other healthy volunteers in study populations consisting mainly of Caucasian and African-American participants [5, 7]. Our study included a group of Caucasian individuals, who received the highest dose of tenapanor (90 mg twice daily for 7 days), primarily to generate additional safety data at the highest dose, including digital ECG measurements. This is important from a global development perspective. This study found no evidence of any clinically significant effects on digital ECG measurements in either the Japanese or the Caucasian individuals receiving tenapanor. The most common adverse events in individuals receiving tenapanor that were judged by the study investigator as being related to treatment were gastrointestinal in nature, with diarrhea being the most frequent event. The occurrence of adverse events such as diarrhea following tenapanor treatment may be expected, given the pharmacological effect that leads to increased water retention in the gastrointestinal tract. This is also reflected in the observation that tenapanor treatment resulted in softer stools compared with placebo in all treatment groups in this study, which is consistent with data from other healthy volunteer studies of tenapanor [5].

This study in Japanese participants was conducted in the USA in Japanese people who had lived outside Japan for less than 5 years. It is difficult to judge whether different results would have been obtained if the study had been conducted in Japan because it is unclear whether these individuals had changed their diet since leaving Japan. Notwithstanding these considerations, all participants received a diet standardized for sodium content during the study, including during the run-in period. No other food items, spices, or additional table salt was allowed, thereby ensuring controlled sodium intake. Although the diet was not standardized for phosphate content, all participants did receive the same meals on the same study days, thereby facilitating consistent phosphate intake.

In conclusion, tenapanor treatment resulted in reductions in absorption of sodium and phosphate from the gut in healthy Japanese volunteers. Tenapanor had minimal systemic exposure, was well tolerated, and produced a softer stool consistency. Further evaluation of tenapanor in different patient groups with hyperphosphatemia in CKD requiring dialysis and patients with constipation-predominant IBS is warranted.

## Electronic supplementary material

Below is the link to the electronic supplementary material.

Supplementary material 1 (PDF 66 kb)
