# Supplementary material for: A phase 1 study of the safety, tolerability, pharmacodynamics, and pharmacokinetics of tenapanor in healthy Japanese volunteers
Source: Clin Exp Nephrol. 2016 Jul 1;21(3):407–16. doi: 10.1007/s10157-016-1302-8 (PMC5486465; doi:10.1007/s10157-016-1302-8)
Supplement: Supplementary file 1 — Supplementary material 1 (PDF 66 kb) [file 10157_2016_1302_MOESM1_ESM.pdf]

A phase 1 study of the safety, tolerability, pharmacodynamics, and pharmacokinetics of tenapanor in healthy Japanese volunteers

Authors

Susanne Johansson, David P. Rosenbaum, Mikael Knutsson, Maria Leonsson-Zachrisson

S. Johansson, M. Knutsson, M. Leonsson-Zachrisson

AstraZeneca Gothenburg, Mölndal, Sweden

D. Rosenbaum

Ardelyx Inc., Fremont, CA, USA

Corresponding author:

David P. Rosenbaum

Ardelyx, Inc.

34175 Ardenwood Blvd, Suite 200

Fremont, CA 94555, USA

Email: drosenbaum@ardelyx.com

Phone: +1 510 745 1752

Supplementary Table S1. Blood pressure and clinical chemistry values for healthy Japanese individuals receiving repeated doses of tenapanor or placebo

		Repeated-dose groups[a]				
		Tenapanor 15 mg b.i.d. (*n* = 12)	Tenapanor 30 mg b.i.d. (*n* = 12)	Tenapanor 60 mg b.i.d. (*n* = 12)	Tenapanor 90 mg b.i.d. (*n* = 12)	Placebo (*n* = 12)
Systolic blood pressure (mmHg)	Baseline	107.4 ± 8.51	109.4 ± 7.25	103.3 ± 10.02	107.2 ± 9.32	106.8 ± 8.47
	Day 8	111.7 ± 12.48	113.2 ± 10.25	104.8 ± 9.69	111.3 ± 13.67	110.3 ± 12.26
Diastolic blood pressure (mmHg)	Baseline	62.1 ± 7.86	63.1 ± 7.84	59.9 ± 5.00	59.7 ± 4.83	60.8 ± 7.43
	Day 8	66.1 ± 7.14	65.5 ± 8.55	61.8 ± 5.80	64.0 ± 8.55	66.1 ± 9.92
Serum sodium (mmol/L)[b]	Baseline	141.3 ± 2.15	140.2 ± 1.19	141.3 ± 1.15	140.9 ± 1.51	139.5 ± 1.81
	Day 8	140.6 ± 1.51	139.4 ± 1.68	139.8 ± 1.36	138.9 ± 0.90	139.6 ± 1.29
Serum phosphorus (mg/dL)[b]	Baseline	3.67 ± 0.440	3.68 ± 0.256	4.03 ± 0.458	4.07 ± 0.416	3.94 ± 0.472
	Day 8	3.88 ± 0.331	3.89 ± 0.211	4.31 ± 0.429	4.29 ± 0.462	4.16 ± 0.492
Serum calcium (mg/dL)[b]	Baseline	9.75 ± 0.421	9.68 ± 0.205	9.76 ± 0.275	9.84 ± 0.275	9.76 ± 0.294
	Day 8	9.68 ± 0.333	9.73 ± 0.299	9.80 ± 0.286	9.62 ± 0.279	9.72 ± 0.340

Data shown are mean ± standard deviation.

[a]Dosing over 7 days.

[b]Placebo, *n* = 11

b.i.d. twice daily
